# Synthetic Teichoic Acid Conjugate Vaccine against Nosocomial Gram-Positive Bacteria

**DOI:** 10.1371/journal.pone.0110953

**Published:** 2014-10-21

**Authors:** Diana Laverde, Dominique Wobser, Felipe Romero-Saavedra, Wouter Hogendorf, Gijsbert van der Marel, Martin Berthold, Andrea Kropec, Jeroen Codee, Johannes Huebner

**Affiliations:** 1 Division of Infectious Diseases, Department of Medicine, University Medical Center Freiburg, Freiburg, Germany; 2 EA4655 U2RM Stress/Virulence, University of Caen Lower-Normandy, Caen, France; 3 Bio-organic Synthesis Unit, Faculty of Science, Leiden Institute of Chemistry, Leiden University, Leiden, Netherlands; 4 Division of Pediatric Infectious Diseases, Dr. von Hauner Children's Hospital, Ludwig-Maximilians-University, Munich, Germany; 5 German Center for Infection Research (DZIF), Partnersite Munich, Munich, Germany; Instituto Butantan, Brazil

## Abstract

Lipoteichoic acids (LTA) are amphiphilic polymers that are important constituents of the cell wall of many Gram-positive bacteria. The chemical structures of LTA vary among organisms, albeit in the majority of Gram-positive bacteria the LTAs feature a common poly-1,3-(glycerolphosphate) backbone. Previously, the specificity of opsonic antibodies for this backbone present in some Gram-positive bacteria has been demonstrated, suggesting that this minimal structure may be sufficient for vaccine development. In the present work, we studied a well-defined synthetic LTA-fragment, which is able to inhibit opsonic killing of polyclonal rabbit sera raised against native LTA from *Enterococcus faecalis* 12030. This promising compound was conjugated with BSA and used to raise rabbit polyclonal antibodies. Subsequently, the opsonic activity of this serum was tested in an opsonophagocytic assay and specificity was confirmed by an opsonophagocytic inhibition assay. The conjugated LTA-fragment was able to induce specific opsonic antibodies that mediate killing of the clinical strains *E. faecalis* 12030, *Enterococcus faecium* E1162, and community-acquired *Staphylococcus aureus* strain MW2 (USA400). Prophylactic immunization with the teichoic acid conjugate and with the rabbit serum raised against this compound was evaluated in active and passive immunization studies in mice, and in an enterococcal endocarditis rat model. In all animal models, a statistically significant reduction of colony counts was observed indicating that the novel synthetic LTA-fragment conjugate is a promising vaccine candidate for active or passive immunotherapy against *E. faecalis* and other Gram-positive bacteria.

## Introduction

The incidence of infections caused by multidrug resistant enterococci has become a worldwide problem over the last decades, particularly in immunocompromised patients [1]. Acquired resistance to β-lactams and vancomycin has spread almost through all patient populations, not only making nosocomial infections caused by this genus extremely difficult to treat, but also highlighting the necessity to develop alternative treatments [2]. Effective immunotherapies are usually directed against virulence factors like capsular polysaccharides that are present on the outside of the bacterial membrane and which often play a role to evade host responses [3]. Our group has previously identified an enterococcal surface antigen, lipoteichoic acid (LTA), present in nonencapsulated *E. faecalis* strains, that is able to induce opsonic antibodies and protect against *E. faecalis* and *E. faecium* bacteremia [4].

Lipoteichoic acids are amphiphilic glycoconjugate polymers and are important constituents of the cell wall of many Gram-positive bacteria such as staphylococci, streptococci, bacilli, clostridia, corynebacteria and listeria [3], [5]. They play crucial roles in cell division, membrane elasticity, porosity and anchoring of surface proteins [3], [6]. The chemical structure of LTAs varies among organisms, but in the majority of Gram-positive bacteria LTA has a relatively conserved poly-1,3-(glycerolphosphate) backbone structure with limited variability, which may be due to its biosynthetic pathway [7], [8]. This backbone represents the shared epitope amongst different bacterial strains and variation of the LTA structures between organisms originates from the type and number of carbohydrate appendages and length of the polyglycerol phosphate chain [3]. The glycolipid anchor of LTA has been reported to be an integral part of the immunostimulatory activity of LTA, although it has also been argued that lipopeptides and lipoproteins that contaminate LTA when isolated from biological sources, are responsible for this activity. The polyglycerol-phosphate backbone has no innate immunostimulatory activity itself and small teichoic acid fragments are poor immunogens [9]–[11]. Polysaccharide antigens that are intrinsically poorly immunogenic [12] are often conjugated to a carrier protein to elicit optimal anti-polysaccharide responses, and to induce humoral immune responses with the characteristics of a T-cell dependent antigen [12], [13]. Synthetic oligosaccharide-protein conjugate vaccines have emerged recently as an interesting strategy in vaccinology, since they offer two major advantages: a well-defined chemical structure (chain length, nature of the epitope, well-established carbohydrate/protein ratio, single type of linkage between the antigen and the carrier) and lack of impurities present in polysaccharides obtained from bacterial cultures [14], [15]. This would apply also for a teichoic acid-based vaccine. The accessibility of the highly conserved LTA polymer on the cell surface, its relatively uniform basic structure and its non-inflammatory nature would be advantages of a synthetic LTA vaccine that targets a wide variety of LTAs in different Gram-positive pathogens. Very recently, a tetanus toxoid conjugate of a 10-mer polyglycerolphosphate (PGP) was evaluated for its potential use as a conjugate vaccine directed against *S. aureus* [11]. Chen and coworkers evaluated the use of this PGP as a prophylactic conjugated vaccine in a mouse model and were able to demonstrate that serum raised against the conjugate elicits specific IgG capable of enhancing in vitro opsonophagocytic killing of *S. aureus* strains and clearance of staphylococcal bacteremia in vivo [11]. Although Chen *et al.* did not evaluate the potential use of this PGP-based conjugate vaccine against other Gram-positive pathogens, they suggest cross-protection against organisms expressing this highly conserved backbone [11].

We have previously shown that opsonic antibodies directed against LTA from *E. faecalis* are cross-reactive against LTA present in *Staphylococcus epidermidis*, *Staphylococcus aureus* and group B streptococci, and that they mainly bind to the poly-1,3-(glycerolphosphate) backbone, suggesting that this minimal structure may be sufficient for vaccine development against some Gram-positive bacteria [8]. We also have previously demonstrated that short synthetic oligoglycerol phosphates are able to absorb up to 91% of the opsonophagocytic killing of serum raised against LTA from *E. faecalis* 12030 against the homologous strain and also against *S. aureus* strains, indicating that these synthetic antigens could be used as templates for vaccine development [8], [16]. In the present study, we evaluated the antigenicity of well-defined synthetic teichoic acid fragments and the development of a synthetic TA-protein conjugate as a vaccine candidate. We show that the semi-synthetic model vaccine modality is capable of eliciting opsonic and protective antibodies that promote *in vitro* opsonophagocytic killing, clearance of bacteria after active and passive immunization and effectively protect against enterococcal bacteremia in a rat endocarditis model.

## Materials and Methods

### Synthesis of Teichoic Acid Fragments

**Figure 1:**
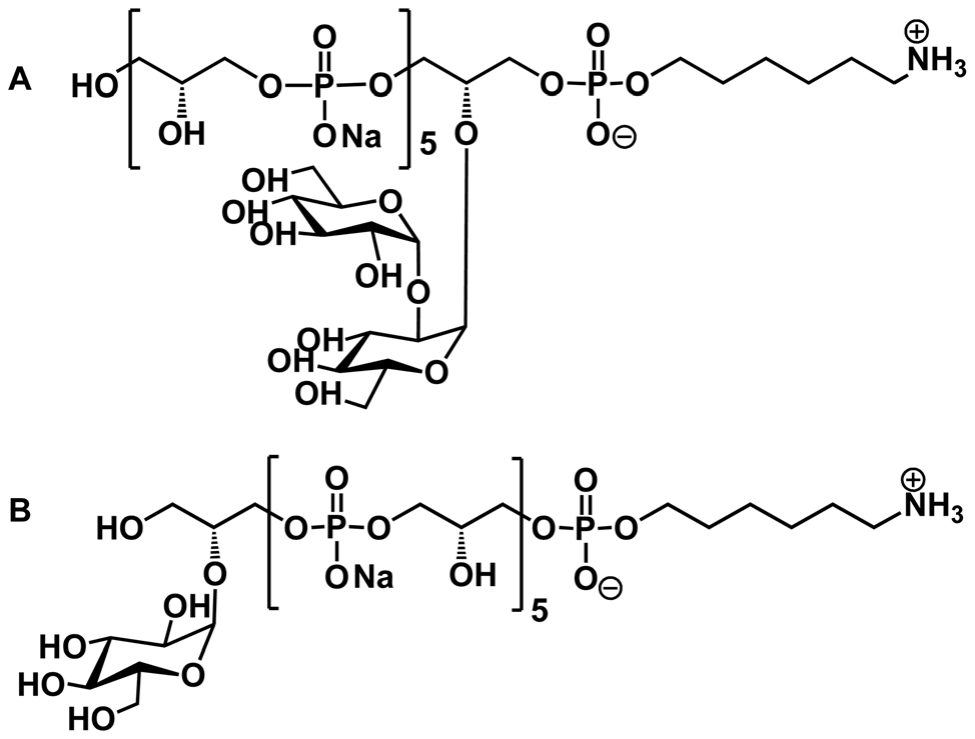
Structure of synthetic teichoic acid hexamers WH5 (A) and WH7 (B).

The short teichoic acid fragments used in this study were synthesized as described earlier using a combination of solution phase, light fluorous supported and automated solid phase techniques [8], [17]–[19]. To scale up the synthesis of the hexaglycerolphosphate with a single glycosyl appendage (WH7, see Figure 1A), a light fluorous supported synthesis was applied [19]. Kojibiosyl [α-Glc-1→2-α-Glc] functionalized hexaglycerol phosphate (WH5, Figure 1B) was assembled using a solution phase synthesis approach [17].

### Synthesis of the Model Teichoic Acid Vaccine

**Figure 2:**
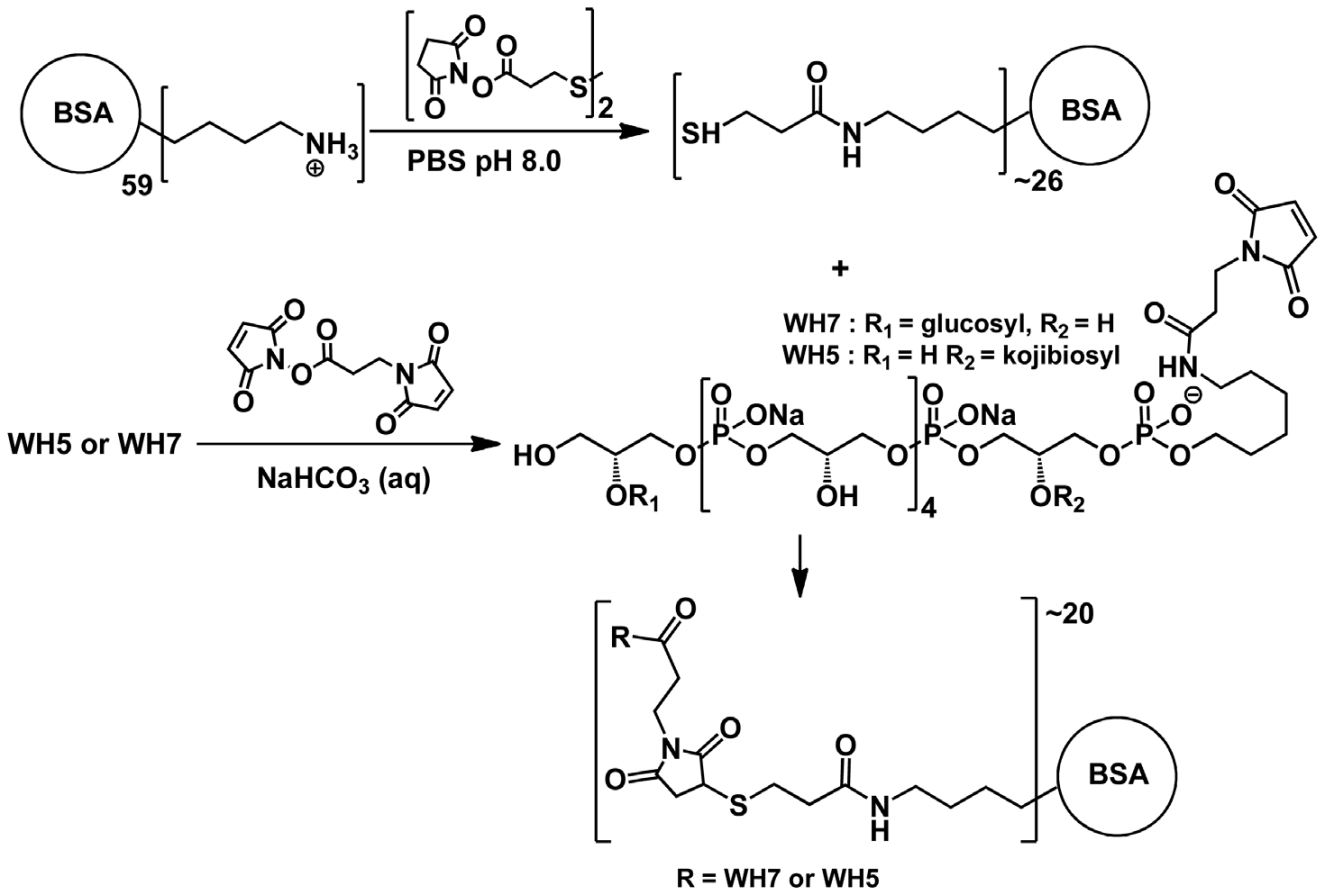
Synthesis of WH5-BSA and WH7-BSA conjugates.

Maleimides derivatives of WH7 and WH5 were obtained by treatment of the glycosylated glycerol phosphate hexamers [16] with *N*-succinimidyl-3-maleimido propionate ester (See Supporting Information S1 for full experimental details and characterization of the new compounds). Bovine serum albumin BSA was thiolated as described by Verez-Bencomo and co-workers [21] by treatment with thiopropionic acid hydroxysuccinimide ester homodisulfide and subsequent reduction of the disulfides. Quantification of the thiols using Ellman's reagent indicated that ±43 thiol groups per BSA molecule were present. The maleimides of WH7 and WH5 were then conjugated with thiolated-BSA through a Michael type addition [20], [21] to give conjugates WH7-BSA and WH5-BSA. The teichoic acid conjugates were purified by dialysis and analyzed by SDS-PAGE, which revealed a broad band for the conjugates around 95 KDa and 100 KDa for the WH7 and WH5-conjugates, respectively (Figure 2). The conjugates were also analyzed on protein and sugar content. Combined, these analyses indicated that approximately 20 teichoic acid fragments per BSA molecule were installed.

### Purification of LTA

Lipoteichoic acid from *E. faecalis* 12030 was obtained by butanol extraction as described elsewhere [6].

### Bacterial Strains and Rabbit Immunizations

The bacterial strains used in this study were the clinical strains *E. faecalis* 12030 [22], *E. faecium* 1162 [23] and community-acquired *S. aureus* MW2 (USA400) [24]. A New Zealand white rabbit was immunized with purified LTA from *E. faecalis* 12030 as described elsewhere (anti-LTA) [4]. Three more rabbits were immunized with synthetic LTA WH5 conjugated with BSA (antiWH5-BSA), synthetic LTA WH7 conjugated with BSA (antiWH7-BSA), and unmodified BSA (anti-BSA). Two subcutaneous injections of 10 µg of conjugate or protein were given 2 weeks apart together with Freund's incomplete antigen; in the fourth and eighth week, three injections of 5 µg were given intravenously; and a final bleed was collected in the ninth week. All sera samples were heat-inactivated at 56°C for 30 min to inactivate complement components. For all experiments, sera from the final bleed of the rabbit were used.

### Measurement of antigen (LTA and WH7)-specific IgG titers in rabbit immune sera

Total IgG concentration was determined in each pre-bleed, test bleed (i.e. after 5th antigen injection) and terminal bleed sera with the Easy-Titer Rabbit IgG Assay kit (Thermo Scientific) according to the manufacturer's instructions. Total IgG concentration in each serum sample was adjusted to 9.3 mg IgG/mL and serum specific IgG titers against WH7 or LTA were measured by ELISA. Nunc-immuno Maxisorp MicroWell 96 well plates were coated either with 0.2 µg of WH7 antigen (previously conjugated with Tetanus Toxoid as described by Hoogerhout and co-workers [25], [26]) or 0.125 µg of LTA from *S. aureus* purchased from Sigma (St. Louis, Mo.) in 0.2M carbonate-bicarbonate coating buffer. Plates were incubated overnight at 4°C, washed three times after incubation with PBS containing 0.05% Tween 20 and blocked with 3% cold water fish skin gelatin (Sigma, St. Lois, Mo.) in PBS at 37°C for 2 hours. Rabbit sera were plated in twofold serial dilutions and incubated 1 hour at 37°C, starting with a dilution of 1∶12.5 for each serum tested. Alkaline-phosphatase-conjugated anti-rabbit IgG produced in goat (Sigma) diluted 1∶1.000 was used as secondary antibody and p-nitrophenyl phosphate (Sigma) was used as substrate (1 mg/mL in 0.1M glycine, 1 mM MgCl_2_, 1 mM ZnCl_2_, pH 10.4). After 60 min of incubation at room temperature, the absorbance was measured at 405 nm on a Tecan Infinite 200 PRO (Tecan Group Ltd.). Each experiment was performed twice at different time-points, and wells were measured in quadruplicate, with a coefficient of variation for each measured sample less than 12%. Serum Ig titers were calculated as follows: For each serum sample, a plot of OD value against the reciprocal of the dilution (Log(1/dilution)) was used to calculate the intercept with the specified cutoff value of each test, and this value was taken as the ELISA end point titer. The value extrapolated from the standard curve was then multiplied by the inverse of that dilution to generate the final inverse titer [27].

### Opsonophagocytic Assay

An in vitro opsonophagocytic assay (OPA) was performed as described elsewhere [28] using baby rabbit serum as complement source and rabbit sera raised against either purified LTA or the conjugated synthetic LTA antigens. White blood cells (WBCs) were freshly prepared from human blood collected from healthy adult volunteers. Bacterial strains were grown to an OD_650 nm_ = 0.400 in tryptic soy broth (TSB). For the assay, the following components were mixed: 100 µl of PMNs; 100 µl of serum dilutions (as indicated), 100 µl of absorbed baby rabbit complement at a dilution of 1∶15 and 100 µl of the bacterial suspension adjusted to the desired colony counts (i.e. 1∶1 relation PMNs/bacteria). The mixture was incubated on a rotor rack at 37°C for 90 min, and samples were plated in duplicate at time 0 and after 90 min. Percent killing was calculated by comparing the colony forming units (CFUs) surviving in the tubes with bacteria, WBCs, complement and antibody, to the CFUs surviving in the tubes with all these components but lacking WBCs. Each experiment was performed at least twice, under the same conditions and with different blood donors, and measured in quadruplicate samples. Variation in the percentage of killing between the two independent experiments was less than 15%, showing the same trend and indicating the reliability of the procedure and the reproducibility of the experiment.

### Opsonophagocytic Inhibition Assay (OPIA)

Synthetic TA mimetics and purified LTA from *E. faecalis* 12030 were used to evaluate its function as inhibitors of the opsonic killing generated by anti-LTA serum at different concentrations (from 0.08 to 100 µg/mL), by incubating them for 60 minutes at 4°C with an equal volume of a 1∶200 dilution of anti-LTA serum or the sera raised against WH7. After this incubation step, the opsonophagocytic assay was performed as described above using the mix inhibitor/anti-LTA or inhibitor/anti-WH7-BSA serum instead of the serum dilutions. Inhibition assays were performed at serum dilutions yielding 70 to 80% of opsonic killing of the inoculum without the addition of the inhibitor. The inhibition of killing was calculated as the percentage of CFUs surviving opsonophagocytic killing when the inhibitor was used compared to those surviving when no inhibitor was present. Each experiment was performed at least twice, under the same conditions and with different blood donors, and measured in quadruplicate samples. Variation in the percentage of killing between the two independent experiments was less than 15%, showing the same trend and indicating the reliability of the procedure and the reproducibility of the experiment.

### Whole cell ELISA

Bacterial strains *E. faecalis* 12030, *E. faecium* E1162 and *S. aureus* MW2, were grown in tryptic soy agar overnight and resuspended in PBS to an OD_650_ of 0.4 (∼5×10^8^ CFU/mL). From each bacterial suspension, 100 µL was added to wells of Nunc-immuno Maxisorp MicroWell 96 well plates and incubated overnight at 4°C. The wells were washed three times with PBS containing 0.05% Tween 20. After washing, the wells were blocked with PBS containing 3% cold water fish skin gelatin (Sigma, St. Louis, Mo.) for 2 h at 37°C. Antibodies normal rabbit serum (NRS), anti-LTA, anti WH5-BSA and anti WH7-BSA were tested at 1∶100 dilution, with final concentration of 93 µg IgG's/mL. Alkaline-phosphatase conjugated anti-rabbit IgG produced in goat (Sigma) diluted 1∶1,000 was used as secondary antibody and p-nitrophenyl phosphate (Sigma) was used as substrate (1 mg/mL in 0.1M glycine, 1 mM MgCl_2_, 1 mM ZnCl_2_, pH 10.4). After 60 min of incubation at room temperature, the absorbance was measured at 405 nm on a Tecan Infinite 200 PRO (Tecan Group Ltd.). Each experiment was performed twice, measured in quadruplicate samples, with a coefficient of variation for each measured sample less than 15%. Both antibodies were diluted in PBS with 1% cold water fish skin gelatin.

### Mouse Sepsis Model – Active Immunization

Six to eight week-old female BALB/c mice (Charles River Laboratories Germany GmbH) were vaccinated with WH7-BSA conjugate or unmodified BSA antigens. Mice were injected subcutaneously with 10 µg of antigen emulsified in 100 µl complete Freund's adjuvant. Eight days post vaccination mice were boosted subcutaneously with 10 µg of antigen emulsified in 100 µl incomplete Freund's adjuvant. On days 14, 16, 18, 32 and 35 mice were boosted intraperitoneally with 5 µg of antigen suspended in saline. Challenge of the vaccinated mice was performed intravenously seven days after final boost with 3×10^7^ CFUs of *E. faecalis* 12030. Mice were sacrificed 24 hours after challenge and colony counts in the liver were determined.

### Mouse Sepsis Model – Passive Immunization

The model was performed as previously described [4]. In brief, six to eight week-old female BALB/c mice (Charles River Laboratories Germany GmbH) were immunized i.p. with 100 µL of anti WH7-BSA serum or normal rabbit serum (NRS). After 24 hours, mice were infected i.v. with 9.4×10^6^ CFUs of *E. faecalis* 12030 via the tail vein. Mice were sacrificed 24 hours after challenge and colony counts in liver were determined.

### Rat Endocarditis Model

A rat endocarditis model was performed as described previously [29]. In brief, female Wistar rats (Charles River Laboratories Germany GmbH), were anesthetized with 5.75% ketamine and 0.2% xylazine. Nonbacterial thrombotic endocarditis was caused by insertion of a plastic catheter (polyethylene tubing; Intramedic PE 10) via the right carotid artery. The catheter was advanced through the aortic valve into the left ventricle, secured properly and left in place. Rats were monitored closely after the catheter implantation. Ten rats were randomly separated in two groups, one received intravenously 500 µL of normal rabbit sera (NRS, Cedarlane, Burlington, Canada) during catheter implantation, and the second group received 500 µL of the anti WH7-BSA serum. An injection of 500 µL of NRS and anti WH7-BSA was made i.p. after 24 hours of catheter implantation and 4 hours after bacterial challenge. An inoculum of 1.18×10^5^ C.F.U.s of *E. faecalis* 12030 was injected via the tail vein in each rat 48 hours after catheter implantation. On postoperative day 6, rats were sacrificed and the correct placement of the catheter was verified. Valve vegetations were removed aseptically, weighed and homogenized in 500 µL TSB. Quantitative assessment was performed by weighing the vegetations as well as culturing serial dilutions on agar plates incubated over night at 37°C. Only animals with correct placement of the catheter were included in the study.

### Statistics

The software program GraphPad PRISM version 5.00 was used for statistical analysis. The percentage of opsonophagocytic killing and absorbance in whole-cell ELISA was expressed as the geometrical mean ± the standard errors of the means and statistical significance was determined by one way ANOVA with Dunnett's post-test for multiple comparisons of groups. For animal models statistical significance was determined by nonparametric t-test with Mann-Whitney post-test and P values ≤0.05 were considered statistically significant.

### Ethics Statement

All animal experiments were performed in compliance with the German animal protection law (TierSchG). The mice and rats were housed and handled in accordance with good animal practice as defined by FELASA and the national animal welfare body GV-SOLAS. The animal welfare committees of the University of Freiburg (Regierungspräsidium Freiburg Az 35/9185.81/G-07/15, Az 35/9185.81/G07-72) approved all animal experiments.

## Results

### Synthetic antigen selection and synthesis of the teichoic acid carrier protein conjugates

**Figure 3:**
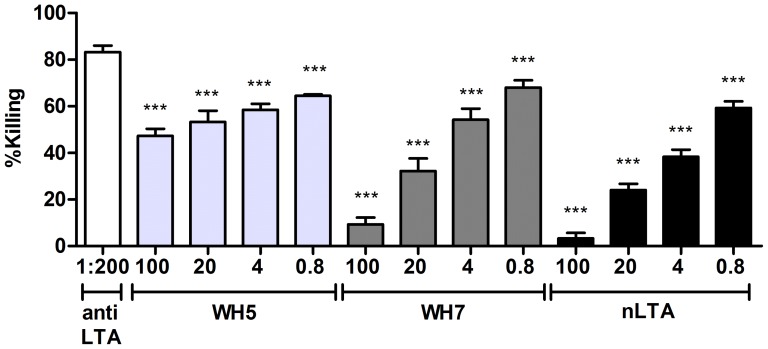
Evaluation of anti-LTA antibodies specificity for synthetic LTA hexamers and native LTA. Antibodies raised against LTA from *E. faecalis* 12030 were evaluated for their ability to bind specifically the different synthetic TA hexamers WH5 and WH7, and native LTA purified from *E. faecalis* 12030 (nLTA). Anti-LTA sera was used at final dilution of 1∶200 and the strain tested was *E. faecalis* 12030. Inhibitors WH5, WH7 and nLTA at concentrations of 100, 20, 4 and 0.8 µg/mL were preincubated with anti-LTA antibodies for 1 hour at 4°C prior OPA. Opsonic killing of the target strain with non-absorbed antibodies was used to assess the reduction of opsonic killing produced by each inhibitor. All inhibitors tested showed statistical significance in comparison to control anti-LTA serum (P value <0.001). The corresponding dilutions of inhibitor used in the OPA are indicated in the x-axis and the % killing in the y-axis. Bars represent the mean of data and the error bars represent the standard error of the mean.

The library of teichoic acid fragments we have synthesized contains fragments varying in length (ranging from 6 to 30 glycerol phosphate monomers), glycosylation pattern (α-glucosyl, α-kojibiosyl, α-glucosamine and α-*N*-acetyl glucosamine) and terminal functionality (either a phosphate or primary alcohol) [8], [16], [17]. From this library we have selected WH7 as an optimal synthetic antigen because of its ability to inhibit the opsonic killing of antibodies raised against LTA from *E. faecalis* 12030 by more than 80% (See Figure 3) [16]. Furthermore, the synthesis of WH7 can be readily scaled to provide enough material allowing the generation of sufficient amounts of the conjugate [19]. Surprisingly we have found that the analogous WH5, featuring an α-kojibiosyl substituent that is found in native *E. faecalis* LTA is devoid of such activity (Figure 3). Therefore, WH5 was chosen as a negative control and both synthetic TA fragments were conjugated to BSA. To this end, the approach used by Verez-Bencomo *et al.* for the development of the Quimihib-vaccine [20] was employed, because of the structural similarities between the Hib ribitol-based polysaccharide and the synthetic TA fragments at hand. We selected BSA as a carrier protein because of its stability, ease of handling, favorable molecular weight, non-glycosylated nature and modification possibilities. The TA fragments, functionalized with a primary amine spacer, were equipped with a reactive maleimide group and conjugated to thiolated BSA as depicted in Figure 2. The obtained conjugates were characterized by SDS-PAGE and analyzed for carbohydrate and protein content, revealing a carbohydrate-protein ratio of ∼1.5∶ 1, indicating that approximately 20 copies of antigen were present per carrier molecule.

### LTA and teichoic acid fragment-specific IgGs are induced after rabbit immunizations

**Figure 4:**
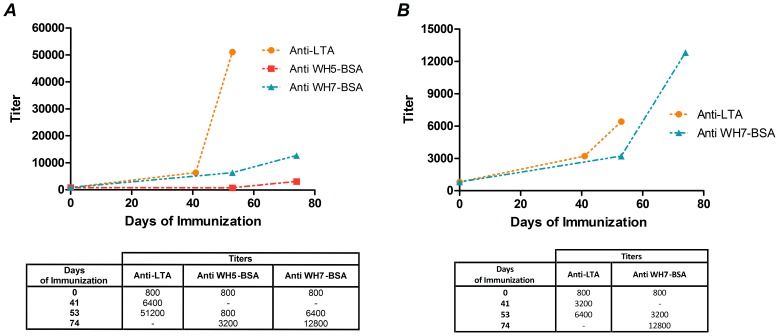
LTA and WH7 -specific IgG antibody titer curves. (**A**) LTA from *S.aureus* specific IgG titers of sera raised against native LTA (Anti-LTA), synthetic WH5-BSA conjugate (Anti WH5-BSA) and synthetic WH7-BSA conjugate (Anti WH7-BSA). (**B**) WH7 specific IgG titers of sera raised against native LTA (Anti-LTA) and synthetic WH7-BSA conjugate (Anti WH7-BSA).

According to figure 4, LTA-specific IgG antibodies were generated during each immunization procedure using either native LTA from *E. faecalis* 12030 (Figure 4A) or WH7-BSA conjugate (Figure 4B). Antibodies raised against the synthetic hexamer WH7 are able to bind and recognize native LTA, but to a lesser extent than antibodies raised against the whole LTA molecule, while only a very low titer is observed for the terminal bleed of anti WH5-BSA serum. A different behavior is observed for WH7 titers of anti-LTA and anti WH7-BSA sera, where higher titers were obtained for anti WH7-BSA serum in comparison to anti-LTA serum.

### Teichoic acid conjugates WH5-BSA and WH7-BSA induced antibodies with opsonic activity

**Figure 5:**
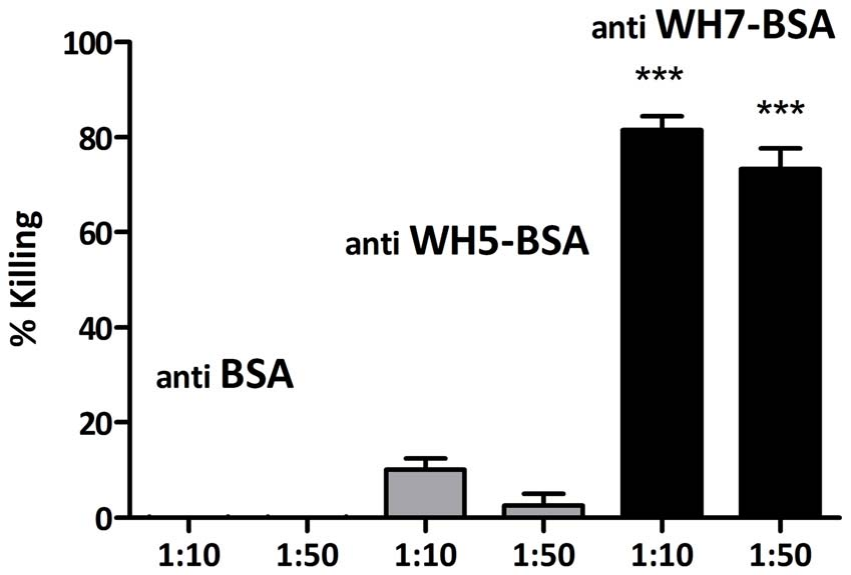
Opsonophagocytic assay. This experiment was used to test the ability of antibodies raised against conjugates with WH5 and WH7, and BSA to mediate killing in the strain *S. aureus* MW2. Anti WH7-BSA sera mediate opsonophagocytic killing of *S. aureus* MW2 in vitro, while the sera anti-BSA and anti-WH5-BSA do not. Comparison of killing percentages of same dilutions (i.e. 1∶10 or 1∶50) with control anti BSA serum, only showed statistical significance for anti WH7-BSA (*** P value <0.001). The different sera and the corresponding dilutions used in the OPA are indicated on the x-axis and the % killing on the y-axis. Bars represent the mean of data and the error bars represent the standard error of the mean.

To confirm that opsonic antibodies were induced by the immunization with the conjugate WH7-BSA but not by immunization with BSA or the WH5-BSA conjugate, the opsonophagocytic activity of the sera was tested by OPA using *S. aureus* MW2 as target strain since this strain was shown to be sensitive to anti-LTA antibodies raised against LTA from *E. faecalis* 12030. At a serum dilution of 1∶10 and 1∶50, the serum raised against BSA was not able to mediate opsonic killing against *S. aureus* MW2. A similar behavior was observed for the WH5-BSA serum, with very low opsonophagocytic killing (<10%). On the other hand, the WH7-BSA serum elicited high opsonophagocytic activity (>60%) for both serum dilutions (Figure 5).

### Opsonic antibodies to WH7-BSA conjugate are specific against the teichoic acid conjugate

**Figure 6:**
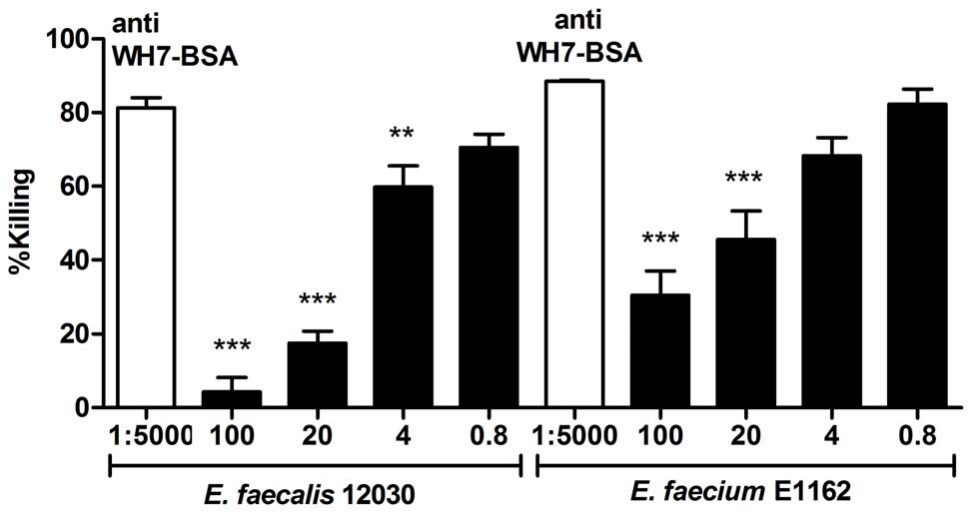
Specificity of anti WH7-BSA serum to WH7-BSA conjugate. Anti WH7-BSA serum was used at final dilution of 1∶5,000 and the strains tested were *E. faecalis* 12030 and *E. faecium* E1162. Inhibitor WH7-BSA conjugate at different concentrations 100, 20, 4 and 0.8 µg/mL was preincubated with anti-LTA antibodies for 1 hour at 4°C prior OPA. Opsonic killing of the target strain with non-absorbed antibodies was used to assess the reduction of opsonic killing produced by each inhibitor, asterisks denote statistical significance (** P<0.01, *** P value <0.001). The corresponding dilutions of inhibitor used in the OPA are indicated on the x-axis and the % killing on the y-axis. Bars represent the mean of data and the error bars represent the standard error of the mean.

To confirm the specificity of the opsonic antibodies to WH7-BSA, an opsonophagocytic inhibition assay (OPIA) was performed by preincubation of anti WH7-BSA serum with a range of dilutions of the synthetic WH7-BSA conjugate. The teichoic acid-conjugate was a potent inhibitor of the opsonophagocytic killing activity of the serum, not only against *E. faecalis* 12030, but also against *E. faecium* E1162 (an *E. faecium* strain sensitive to anti-LTA antibodies). In all cases, the inhibition of anti WH7-BSA antibodies is directly proportional to the amount of inhibitor added (Figure 6).

### Opsonic antibodies to WH7-BSA conjugate are cross-reactive

**Figure 7:**
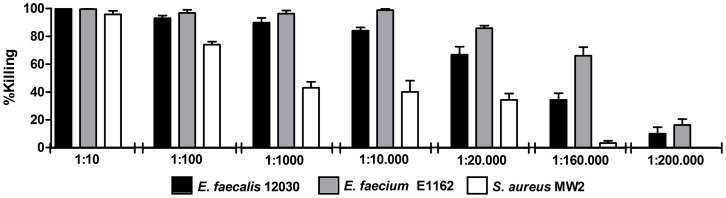
Opsonophagocytic killing activity of anti WH7-BSA antibodies against Gram-positive bacterial strains. The antibodies raised against WH7-BSA conjugate were used at different dilutions 1∶10, 1∶100, 1∶1000, 1∶10.000, 1∶20.000, 1∶160.000 and 1∶200.000, as shown in the x-axis, to assess % killing activity of the target strains *E. faecalis* 12030, *E. faecium* E1162 and *S. aureus* MW2. Bars represent the mean of data and the error bars represent the standard error of the mean.

To explore the specificity of the antibodies directed to the WH7-BSA conjugate for the LTA expressed by Gram-positive bacteria, the opsonic activity towards the strains *E. faecium* E1162, *S. aureus* MW2 and *E. faecalis* 12030 was evaluated. Different concentrations of the serum were tested to titer out the opsonic killing activity of the antibodies. Although it was observed that anti WH7-BSA serum can mediate opsonic killing of all strains tested (Figure 7), the enterococcal strains are effectively killed at higher dilutions than *S. aureus* MW2.

### Antibodies directed against LTA and WH7-BSA conjugate bind bacterial cells

**Figure 8:**
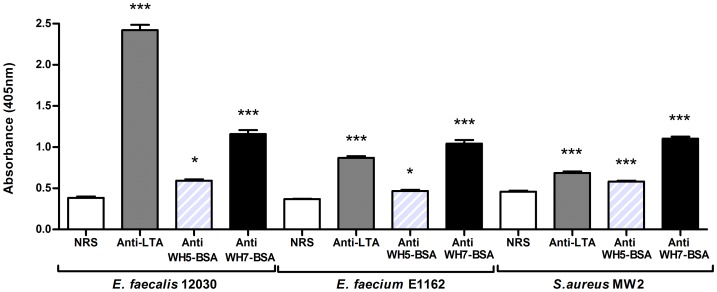
Whole-cell ELISA. Whole cell ELISA of *E. faecalis* 12030, *E. faecium* E1162 and *S.aureus* MW2 using normal rabbit serum (NRS- white bar), anti-LTA serum (grey bar), anti WH5-BSA serum (grey diagonally-striped bar) and anti WH7-BSA serum (black bar). Statistical significance performed by one way ANOVA with Dunnett's post-test for multiple comparison of groups, is denoted in asterisks (* P<0.05, ** P<0.01, *** P<0.001) and was calculated comparing the means of absorbance for each anti sera sample with serum control (NRS).

Immunoreactivity of tested sera against whole bacterial cells was analyzed by whole-cell ELISA (Figure 8). Immune sera anti-LTA, anti WH5-BSA and anti WH7-BSA showed statistically significant binding to cells of *E. faecalis* 12030, *E. faecium* E1162 and *S. aureus* MW2, in comparison with control serum (NRS) (* P<0.05, *** P<0.001). Anti-LTA serum showed stronger signal binding to *E. faecalis* 12030 cells, while anti WH5-BSA serum elicited poor binding to *E. faecalis* and *E. faecium* cells. Serum raised against WH7-BSA-conjugate bound well to all bacterial strains tested.

### Vaccination with WH7-BSA conjugate and immunization with anti WH7-BSA serum promotes clearance of bacteria in mice

**Figure 9:**
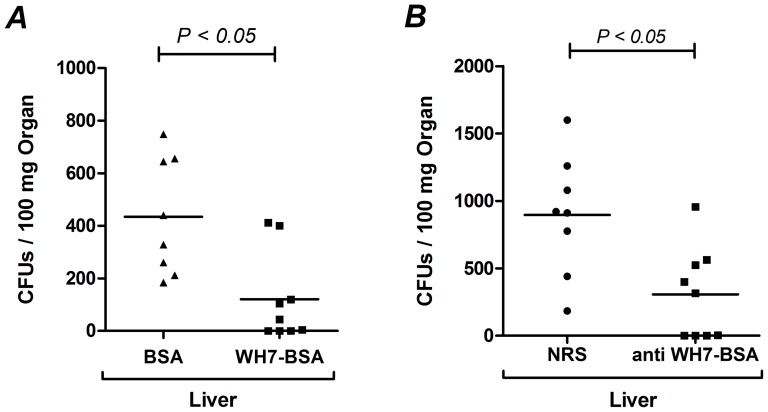
Mouse Sepsis Models. Active Immunization with WH7-BSA conjugate promotes clearance of *E. faecalis* 12030 in liver in comparison with vaccination with unmodified BSA (**A**). Passive immunization with anti WH7-BSA serum facilitates clearance of *E. faecalis* in liver in comparison with normal rabbit serum (NRS) (**B**). After 24 hours of challenging mice were killed, kidney and liver were removed to assess viable counts. Statistical analysis was done by Students t-test, horizontal bars represent geometric means, and a P value <0.05 was considered statistically significant.

To demonstrate protection by the teichoic acid conjugate, active immunization with the purified teichoic acid conjugate was performed in mice. After 2 injections of 10 µg/mouse and 5 injections of 5 µg/mouse, animals showed statistically significantly reduced numbers of *E. faecalis* 12030 in livers 24 hours after inoculation (Figure 9A). In another set of experiments, prophylactic treatment with antibodies raised against the WH7-BSA conjugate significantly reduces bacterial counts in mice. Application of 100 µl anti WH7-BSA serum per mouse 24 hours before bacterial challenge resulted in less bacteria (reduction by factor 5) at 24 hours after inoculation (Figure 9B).

### Opsonic antibodies to WH7-BSA conjugate are protective in a rat enterococcal endocarditis model

**Figure 10:**
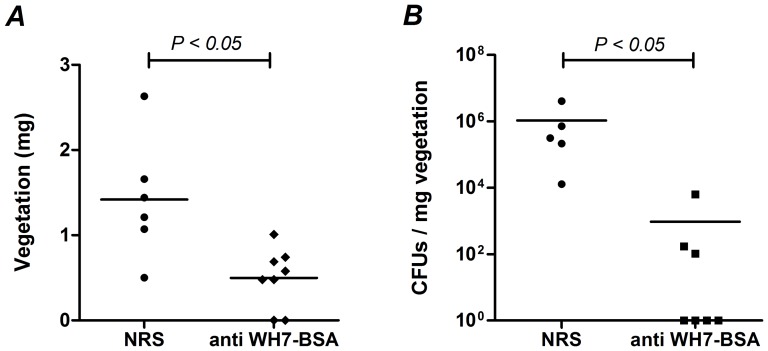
Passive immunization with anti WH7-BSA serum protects rats against enterococcal endocarditis. Thirteen female Wistar rats were catheterized, passively immunized 2 times with 500 µL of anti WH7-BSA sera and NRS, and challenged after 48 hours with 1.18×10^5^ CFUs of *E. faecalis* 12030. After challenging, rats were boosted with either 500 µL of NRS or anti WH7-BSA sera. Three days after challenging, animals were sacrificed and valve vegetations were weighed and quantified. Horizontal lines indicate the geometric means of weighed valve vegetations (**A**) and CFUs per mg of vegetation of *E. faecalis* 12030 (**B**). Significant P values are indicated on the graphs.

To explore if the opsonophagocytic activity of anti WH7-BSA antibodies correlated with an *in vivo* infection model, a rat endocarditis model was performed. Passive immunization with 3 doses of 500 µL of anti WH7-BSA antibodies resulted in dramatic reductions of CFUs and also a significant reduction in valve vegetations (P<0.05) compared to controls (Figure 10); anti WH7-BSA serum mediated complete clearance of *E. faecalis* 12030 in more than 50% of the rats, while all control rats showed endocarditis.

## Discussion

The use of polysaccharide-conjugate vaccines has rapidly emerged as a suitable strategy to combat different pathogenic bacteria. It includes the development of effective vaccines against *Haemophilus influenzae* type b, *Streptococcus pneumoniae*, *Neisseria meningitidis*, *Salmonella typhi* and *S. aureus* [13]–[15], [30]. Many of these approaches have focused on targeting different serotypes by the identification of conserved surface carbohydrate structures. LTAs from different common pathogens have been evaluated as possible vaccine candidates against bacterial infections. Intranasal co-administration of LTA from *Streptococcus pyogenes* with cholera toxin subunit B had induced good pharyngeal IgA and systemic IgGs, suggesting its use as an effective approach in prevention of tonsillitis [31]. Rabbit sera raised against conjugated LTA from *Clostridium difficile* have shown immunoreactivity with cells and spores from other *C. difficile* strains; however, no protection studies have been conducted [32]. In a different approach, Goldenberg and coworkers have demonstrated the ability of human antibodies directed against the phosphorylcholine to protect mice from a lethal dose of *S. pneumonia*. This antigenic component of LTA from *S. pneunomiae* and of the lipopolysaccharide from *H. influenzae* has been proposed as a vaccine candidate against pathogenic bacteria residing the upper respiratory tract [33].

Various synthetic approaches have been reported towards the assembly of well-defined teichoic acid structures of different pathogens, including *Clostridium difficile* [34], [35], *E. faecalis* [7], [8] and *S. aureus* [11], [19]. Martin *et al.* have described the synthesis of oligomers of the LTA antigen in *C. difficile* and used these fragments to define epitopes for vaccine development based on interactions of anti-LTA antibodies with bacteria in the blood of patients. However, no protective efficacy of antibodies directed against the synthetic oligomers has been reported so far [35]. To date, only a chimeric monoclonal antibody developed against LTA from *S. aureus* has been used in clinical trials. Pagibaximab has shown efficacy promoting staphylococcal phagocytosis and survival of animals challenged with coagulase-negative straphylococci [36], [37]. This vaccine appeared safe and well tolerated in healthy adult volunteers and very-low-weight neonates; however, no clear protection has been observed against staphylococcal sepsis in this setting [36]–[39].

Despite the already demonstrated importance of specific antibodies directed against LTA in the treatment of infections caused by many Gram-positive pathogens, the protective efficacy of human pre-existing antibodies, either against LTA structures or phosphorylcholine, is still questionable [33], [40]. Hufnagel and coworkers attribute this fact to the low specificity of pre-existing human LTA antibodies and their non-opsonic nature [40]. Others have explain this biological behavior on the basis of individual variation in IgG subclasses, differences in affinity or avidity, or low specificity of naturally acquired antibodies [33], [41], [42].

We have shown previously that the cross-reactive epitope of anti-LTA antibodies is the poly-1,3-(glycerolphosphate) backbone of LTA in staphylococcal, streptococcal and enterococcal strains; likewise, their ability to opsonize and protect against *E. faecalis*, *E. faecium*, *S. aureus* and *S. epidermidis* bacteremia has been demonstrated [4], [6], [8], [43]. In the current study, two synthetic TA hexamers with different glycosyl substituents were chosen according to their ability to absorb out opsonic anti-LTA antibodies, and their immunogenicity was evaluated after conjugation with a carrier protein. It was found that the kojibiosyl-functionalized TA-conjugate proved ineffective in eliciting opsonic antibodies, which seems to indicate that the kojibiosyl moiety present in the WH5 hexamer probably masks the polyglycerolphosphate backbone. Indeed, we have previously found that the kojibiosyl hexaglycerolphosphate WH5 is unable to inhibit opsonophagocytic killing of *E. faecalis* by serum raised against LTA [8]. On the other hand, the ability of hexamer WH7 to inhibit, in a dose-dependent fashion, the opsonic killing activity of anti-LTA antibodies to *E. faecalis* 12030 was confirmed in agreement with previous findings [16]. The conjugate WH7-BSA was able to induce high-titered opsonic and specific antibodies, showing cross-reactivity with two other pathogenic Gram-positive species. The lower opsonophagocytic killing observed in the staphylococcal strain may be explained by the ability of capsular polysaccharides present in the cell wall of some Gram-positive strains to mask components in the cell wall [8], [44], [45]. Additionally, the opsonic killing activity of the anti WH7-BSA serum observed by OPA, correlates with the ability of the TA conjugate antigen to induce antibodies that promotes clearance of bacteria in the liver of mice, either by active or passive immunization and to protect rats against enterococcal endocarditis by passive immunization. The active immunization schedule used here was chosen because pilot experiments confirmed effective induction of high titers of opsonic antibodies. However, since we did not measure titers against the antigens after all the different injections we cannot assess how many applications of antigen would be necessary to achieve protective titers.

The high affinity against LTA observed in the serum raised against native LTA from *E. faecalis* 12030 is probably caused by the diversity of antibodies against different epitopes of the molecule. Although antibodies directed against the WH7-BSA conjugate show lower binding than anti-LTA serum, these antibodies may have a higher affinity for the specific antigenic determinant present in the WH7 structure that represents the protective epitope, enabling the sera to mediate better in vitro opsonophagocytosis and protect animals against bacteremia or endocarditis. Antibodies directed against different epitopes (i.e. alanine residues present in native LTA but absent in WH7) that are raised through immunization with native LTA do not bind to the synthetic molecule, explaining the relatively low binding of serum raised against native LTA to WH7. The results from the whole cell ELISA confirm these findings since antibodies directed against WH7-BSA are able to bind bacterial cells of *E. faecalis* 12030, *E. faecium* E1162 and *S. aureus* MW2, showing that there is a direct relationship between the antibodies bound to the bacterial cell surface and the ability of antibodies to mediate *in vitro* opsonophagocytic killing or provide protection against bacteremia and endocarditis in animal studies.

In summary, the data presented here describe the potential use of a synthetic polyphosphoglycerol conjugate as vaccine candidate in enterococci. To this end we have used a potent glycerol phosphate hexamer, WH7, selected from a focused library of synthetic TA structures, and conjugated this to BSA, a model carrier protein. We were able to demonstrate that the WH7-BSA conjugate induces specific and opsonic antibodies that promote clearance of *E. faecalis* in the liver of mice and protect against enterococcal endocarditis, suggesting that this teichoic acid conjugate is a promising vaccine candidate against enterococcal infections. Its use as a prophylactic vaccine will require further studies of cross-reactivity against other enterococcal strains and other Gram-positive bacterial species as well as different infection models.

## Supporting Information

Supporting Information S1 **Materials, general methods and NMR spectra of LTA-fragment conjugates synthesis.**
(DOCX)
