# Supplementary material for: Synthetic Teichoic Acid Conjugate Vaccine against Nosocomial Gram-Positive Bacteria
Source: PLoS One. 2014 Oct 21;9(10):e110953. doi: 10.1371/journal.pone.0110953 (PMC4205086; doi:10.1371/journal.pone.0110953)
Supplement: Supporting Information S1 — Materials, general methods and NMR spectra of LTA-fragment conjugates synthesis. (DOCX) [file pone.0110953.s001.docx]

**- Supporting Information -**

**Synthetic Teichoic Acid Conjugate Vaccine Against Nosocomial Gram-positive Bacteria**

Diana Laverde^1,2^, Dominique Wobser^1^, Felipe Romero-Saavedra^1,2^, Wouter Hogendorf^3^, Gijsbert van der Marel^3^, Martin Berthold^1^, Andrea Kropec^1^, Jeroen Codee^3,#^, Johannes Huebner^1,4,5,#^

^1^ *Division of Infectious Diseases, Department of Medicine, University Medical Center Freiburg, Freiburg, Germany.*

^2^ *EA4655 U2RM Stress/Virulence, University of Caen Lower-Normandy, Caen, France.*

^3^ *Bio-organic Synthesis Unit, Faculty of Science, Leiden Institute of Chemistry, Leiden University, Leiden, Netherlands.*

^4^ *Division of Pediatric Infectious Diseases, Dr. von Hauner Children's Hospital, Ludwig-Maximilians-University, Munich, Germany.*

^5^ *German Center for Infection Research (DZIF), Partnersite Munich, Munich, Germany.*

*^#^ shared corresponding authorship*

**Supporting Information S1.** Materials, general methods and NMR spectra of LTA-fragment conjugates synthesis.

**

WH5-maleimide**

To a solution of WH5 (10.0 mg, 6.42 μmol) in a mixture of saturated aqueous NaHCO_3_ (300 μl) and H_2_O (300 μl) was added *N*-succinimidyl-3-maleimido propionate (23.3 mg, 87.4 μmol, dissolved in 1.0 ml 3/2 MeCN/1,4-dioxane). After stirring overnight at RT, the mixture was diluted with H_2_O (~5 ml) and washed with EtOAc (20 ml). The organic layer was extracted once with 5 ml of H_2_O and the combined aqueous layers concentrated under reduced pressure until ~1 ml was left. This solution was applied to a size exclusion column (Sephadex HW40, eluent: 0.05 M Et_3_NHOAc) after which the fractions corresponding to WH5-maleimide were evaporated and, subsequently, lyophilized three times. The crude product (in ~0.5 ml H_2_O) was then eluted through a small column containing Dowex Na^+^ cation-exchange resin (type: 50WX4-200, stored on 0.5 M NaOH in H_2_O, flushed with H_2_O and MeOH before use). After concentration *in vacuo* the purified product was coevaporated three times with 1% AcOH (~5 ml) in order to regain the maleimide in the non-hydrolyzed form. Finally, after lyophilization WH5-maleimide (9.11 mg, 5.52 μmol, 86%) was obtained as an amorphous off-white solid. ^31^P NMR (161.7 MHz, D_2_O, hydrolyzed maleimide): δ = 0.9 (1P), 1.1 (1P), 1.2 - 1.3 (3P), 1.3 (1P); ^1^H NMR (600 MHz, D_2_O, hydrolyzed maleimide): δ = 1.25 - 1.33 (m, 4H, 2 x CH_2_ hexylspacer), 1.41 - 1.47 (m, 2H, CH_2_ hexylspacer), 1.54 - 1.59 (m, 2H, CH_2_ hexylspacer), 2.39 (t, 2H, *J* = 6.6 Hz, CH_2_ maleimidopropionyl), 3.10 (t, 2H, *J* = 6.9 Hz, CH_2_-N hexylspacer), 3.34 - 3.42 (m, 4H, 2 x H-4, CH_2_-N maleimidopropionyl), 3.49 - 3.56 (m, 2H, CH*H* glycerol, H-2), 3.59 - 3.63 (m, 2H, CH*H* glycerol, H-2), 3.67 - 4.01 (m, 37H, 5 x CH glycerol, 11 x CH_2_ glycerol, CH_2_-O hexylspacer, 2 x H-3, 2 x H-5, 4 x H-6), 4.10 - 4.15 (m, 1H, CH glycerol), 5.10 (d, 1H, *J* = 3.7 Hz, H-1), 5.39 (d, 1H, *J* = 3.5 Hz, H-1), 5.84 (d, 1H, *J* = 12.3 Hz, CH maleimide), 6.25 (d, 1H, *J* = 12.3 Hz, CH maleimide); HRMS (hydrolyzed form): C_43_H_84_N_2_O_45_P_6_ + Na^+^ requires 1557.2664, found 1557.2677.

**

WH5-BSA**

To WH5-maleimide (3.4 mg, 2.0 μmol) was added a solution of freshly prepared thiolated BSA (46 nmol protein, max 2.0 μmol thiol, ~3.1 mg) in phosphate buffer saline (PBS, pH 7.2, 1.39 ml). After stirring for 20 hrs at RT, 3-maleimidopropionic acid (0.34 mg, 2.0 μmol, in 100 μl PBS) was added to cap any remaining thiols. After overnight reaction the solution was transferred to a Slide-A-Lyzer dialysis cassette (20 kDa MW cut off, capacity 3 ml) and dialyzed overnight versus PBS (3.0 l) and milliQ (2 x 3.0 l, overnight). The resulting solution was lyophilized giving WH5-BSA (5 mg) as an amorphous white solid. The protein:sugar ratio was found ~1.5:1 and was determined by UV_280_ and a phenol/H_2_SO_4_ assay [S1]. SDS-PAGE analysis shows a broad band around 100 kDa (see Figure S1, gel A, lane 5-7). These combined results indicate the presence of ~20 kojibiosyl TA fragments per molecule of BSA. Details SDS-PAGE (see also figure 1, gel A); SDS-PAGE was performed using 7.5% polyacrylamide gels on which BSA, thiolated BSA, WH5-BSA and Dual Color Marker mixture (containing 37, 50, 75, 100, 150 and 250 kDa markers) were applied after boiling in sample buffer for 5 minutes. Stacking onto the running gel was performed in 30 minutes at 90V. Run time 90-120 minutes at 120V. Visualization of proteins and conjugates was achieved after staining overnight with Coomassie Brilliant Blue followed by washing three times with milliQ over a period of 24 hrs. Lane 1: BSA (600ng), lane 2: BSA (300 ng) and thiolated BSA (500 ng), lane 3: thiolated BSA (600 ng), lane 4: thiolated BSA (1000 ng), lane 5: thiolated BSA (600 ng) and WH5-BSA (600 ng), lane 6: WH5-BSA (600 ng), lane 7: WH5-BSA (1200 ng), lane 8: dual color mix.

**

WH7-maleimide**

To a solution of WH7 (7.0 mg, 5.3 μmol) in a mixture of saturated aqueous NaHCO_3_ (100 μl) and H_2_O (300 μl) was added *N*-succinimidyl-3-maleimido propionate (28.4 mg, 107 μmol, dissolved in 800 μl 3/2 MeCN/1,4-dioxane). After stirring overnight at RT, the mixture was diluted with H_2_O (~5 ml) and washed with EtOAc (20 ml). The organic layer was extracted once with 5 ml of H_2_O and the combined aqueous layers concentrated under reduced pressure until ~1 ml was left. This solution was applied to a size exclusion column (Sephadex HW40, eluent: 0.05 M Et_3_NHOAc) after which the fractions corresponding to WH7-maleimide were evaporated and, subsequently, lyophilized three times. The crude product (in ~0.5 ml H_2_O) was then eluted through a small column containing Dowex Na^+^ cation-exchange resin (type: 50WX4-200, stored on 0.5 M NaOH in H_2_O, flushed with H_2_O and MeOH before use). After concentration *in vacuo* the purified product was coevaporated three times with 1% AcOH (~5 ml) in order to regain the maleimide in the non-hydrolyzed form. Finally, after lyophilization TA WH7-maleimide (5.3 mg, 3.6 μmol, 67%) was obtained as an amorphous off-white solid. ^31^P NMR (161.7 MHz, D_2_O, hydrolyzed maleimide): δ = 1.2 - 1.3 (5P), 1.4 (1P); ^1^H NMR (600 MHz, D_2_O, hydrolyzed maleimide): δ = 1.23 - 1.33 (m, 4H, 2 x CH_2_ hexylspacer), 1.39 - 1.45 (m, 2H, CH_2_ hexylspacer), 1.52 - 1.58 (m, 2H, CH_2_ hexylspacer), 2.38 (t, 2H, *J* = 6.6 Hz, CH_2_ maleimidopropionyl), 3.09 (t, 2H, *J* = 6.9 Hz, CH_2_-N hexylspacer), 3.33 (at, 1H, *J* = 9.5 Hz, H-4), 3.39 (t, 2H, *J* = 6.6 Hz, CH_2_-N maleimidopropionyl), 3.46 (dd, 1H, *J* = 3.9 Hz, 9.9 Hz, H-2), 3.65 - 4.00 (m, 36H, H-3, H-5, H-6, H-6’, CH_2_-O hexylspacer, 6 x CH glycerol, 12 x CH_2_ glycerol), 5.10 (d, 1H, *J* = 3.8 Hz, H-1), 5.83 (d, 1H, *J* = 12.2 Hz, CH maleimide), 6.24 (d, 1H, *J* = 12.3 Hz, CH maleimide); HRMS: C_37_H_72_N_2_O_39_P_6_ + H^+^ requires 1355.2211, found 1355.2225.

**

WH7-BSA**

To a solution of freshly prepared thiolated BSA (68 nmol protein, max 2.9 μmol thiol, ~4.7 mg) in phosphate buffer saline (PBS, pH 7.2, 1.65 ml) was added WH7-maleimide (5.2 mg, 3.5 μmol, in 400 μl PBS). After stirring for 20 hrs at RT, 3-maleimidopropionic acid (0.65 mg, 3.8 μmol, in 150 μl PBS) was added to cap any remaining thiols. After overnight reaction the solution was transferred to a Slide-A-Lyzer dialysis cassette (20 kDa MW cut off, capacity 3 ml) and dialyzed overnight versus PBS (3.0 l) and milliQ (2 x 3.0 l, overnight). The resulting solution was lyophilized giving WH7-BSA (8 mg) as an amorphous white solid. The protein-sugar ratio was found ~1.5:1 and was determined by UV_280_ and a phenol/H_2_SO_4_ [S1]. SDS-PAGE analysis shows a broad band around 95 kDa (see Figure 1, gel B, lane 8,9). These combined results indicate the presence of ~20 TA fragments per molecule of BSA. Details SDS-PAGE (see also Figure S1, gel B); SDS-PAGE was performed using 7.5% polyacrylamide gels on which BSA, thiolated BSA, WH7-BSA and Dual Color Marker mixture (containing 37, 50, 75, 100, 150 and 250 kDa markers) were applied after boiling in sample buffer for 5 minutes. Stacking onto the running gel was performed in 30 minutes at 90V. Run time 90-120 minutes at 120V. Visualization of proteins and conjugates was achieved after staining overnight with Coomassie Brilliant Blue followed by washing three times with milliQ over a period of 24 hrs. Lane 1: BSA (600ng), lane 2: dual color mix, lane 3: BSA (300 ng) and thiolated BSA (500 ng), lane 4: thiolated BSA (500 ng), lane 5: thiolated BSA (1000 ng), lane 6: thiolated BSA (500 ng), lane 7: dual color mix, lane 8: WH7-BSA (1200 ng), lane 9: WH7-BSA (2400 ng).

**
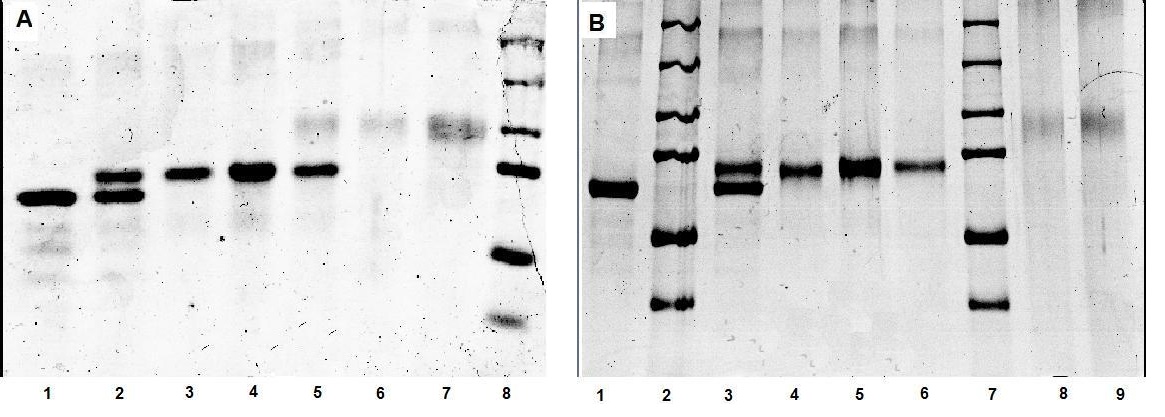
Figure S1.** SDS-PAGE followed by Coomassie Brilliant Blue staining of conjugates WH5-BSA (A) and WH7-BSA (B). A: BSA (lane 1,2), 10 (lane 2-5), conjugate 4 (lane 5-7), DC-marker (lane 8). B: BSA (lane 1,3), 10 (lane 3-6), conjugate 3 (lane 8,9), DC-marker (lane 2,7; top to bottom: 250, 150, 100, 75, 50, 37 kDa).

***References***

S1. Fournier E (2001) Current Protocols in Food Analytical Chemistry. New York, USA: John Wiley & Sons Inc.
